# The Effect of Aqueous Extract of *Rosa canina* on Antioxidant Activity, Sensory Evaluation, and Physicochemical Properties of Yogurt

**DOI:** 10.1002/fsn3.71198

**Published:** 2025-11-12

**Authors:** Shokoofeh Hosseini, Fatemeh Gheitanchi, Abolfazl Kamkar, Sina Sakhaeifar

**Affiliations:** ^1^ Department of Food Hygiene and Quality Control, Faculty of Veterinary Medicine University of Tehran Tehran Iran

**Keywords:** antioxidant activity, dairy products, food quality, natural extracts, sensory evaluation, yogurt

## Abstract

In recent years, there has been growing scientific interest in developing functional foods with medicinal properties. In the context of dairy product innovation, the aqueous extract of *Rosa canina* ( *R. canina* ) has emerged as a promising bioactive compound. This study aimed to evaluate the impact of incorporating *R. canina* aqueous extract at four concentrations (0%, 0.5%, 1%, and 1.5%) on the antioxidant activity, sensory evaluations, and physicochemical properties of yogurt. Antioxidant activity was evaluated using DPPH and ABTS assays, phenolic compounds were quantified with the Folin–Ciocalteu method, physicochemical properties (pH, acidity, syneresis) were measured by standard procedures, and sensory evaluation was performed using a 5‐point hedonic scale. The findings revealed that the addition of *R. canina* extracts significantly enhanced the acidity of the yogurt samples (*p* < 0.05). This value exhibited a pronounced increase from 0.62 to 0.90 g/L in the 1.5% samples over the storage period. A significant reduction in pH was observed across all treatment groups supplemented with *R. canina* extract throughout the storage duration (*p* < 0.05). Furthermore, the incorporation of the extract led to a marked increase in phenolic compounds, with the yogurt containing 1.5% extract reaching a phenolic content of 857.67 (mg GAE eq.)/kg. The antioxidant activity of the yogurt was also significantly enhanced by the addition of the extract compared to the control group (*p* < 0.05), which was increased to 2570.67 (mg BHT eq.)/kg in the 1.5% samples over a 3‐week storage period. However, the syneresis of the yogurt samples increased with higher concentrations of the extract across all storage intervals. The sensory evaluation demonstrated that the inclusion of *R. canina* extracts improved the sensory scores for the appearance, taste, texture, and overall acceptability of the yogurt samples compared to the control. These results suggest that *R. canina* aqueous extract can serve as a functional ingredient in yogurt production, enhancing its bioactive properties and sensory appeal while influencing its physicochemical characteristics.

## Graphical abstract

Addition of aqueous extract of *Rosa canina* to yogurt enhanced phenolic content and antioxidant activity, improved sensory properties, and increased acidity; however, higher extract concentrations also led to greater syneresis, indicating a balance between functional benefits and product stability.

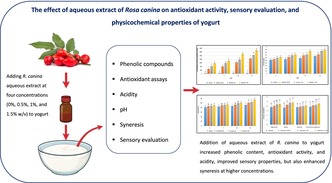

## 1 Introduction

In contemporary times, the utilization of food sources that confer protective benefits against diseases and environmental stressors has gained considerable importance (Angelov et al. 2006; Arendt and Dal Bello 2011; Siró et al. 2008). Yogurt, a widely consumed dairy product, is particularly valued as a fermented food due to its nutritional properties, increased diversity resulting from industrialization, and widespread accessibility (Lourens‐Hattingh and Viljoen 2001; Bourlioux and Pochart 1988). Furthermore, there is a growing trend toward incorporating plant extracts and essential oils as alternatives to synthetic and chemical additives in food products.

Iran, with its diverse climatic conditions, is recognized as one of the world's richest reservoirs of plant biodiversity. Given the favorable taste, nutritional, and therapeutic properties of certain indigenous plants, they hold significant potential for application in the formulation of various functional food products (Krishnaiah et al. 2011). Among these, *Rosa canina* (commonly known as dog rose) is a naturally occurring plant that has been extensively utilized in traditional medicine due to its health‐promoting properties. The fruit of *R. canina* is a rich source of bioactive compounds, including pectin, tannins, carotenoids, flavonoids, and fatty acids, as well as proteins, starches, vitamins, sterols, and minerals, making it a valuable resource for food and pharmaceutical industries. The fatty acid profile of *R. canina* includes caproic acid, caprylic acid, capric acid, undecanoic acid, myristic acid, palmitic acid, palmitoleic acid, heptadecanoic acid, stearic acid, elaidic acid, oleic acid, linoleic acid, linolenic acid, arachidonic acid, and nervonic acid. Notably, this plant is particularly abundant in unsaturated fatty acids, carbohydrates, vitamins A, B, and C, essential minerals, phytosterols, antioxidants, and phenolic compounds (Ercisli 2007). Phenolic compounds represent a significant class of plant‐derived secondary metabolites that play a crucial role in mitigating environmental stresses and protecting biological tissues against oxidative damage induced by free radicals and reactive oxygen species. These compounds are known to prevent the onset of various chronic diseases, including inflammatory disorders, cancer, diabetes, cardiovascular diseases, Alzheimer's disease, and Parkinson's disease. Their antioxidant activity stems from the presence of hydroxyl groups, which enable them to act as electron or hydrogen donors, effectively neutralizing free radicals (Krishnaiah et al. 2011). The antioxidant capacity of phenolic compounds is positively correlated with their concentration, and high molecular weight phenolic compounds exhibit superior free radical scavenging abilities, which are largely influenced by the number of aromatic rings and the structural characteristics of their hydroxyl groups. Due to their diverse chemical structures, phenolic compounds demonstrate varying degrees of antioxidant activity (Salganik 2001).

In a related study, Illupapalayam et al. (2014) investigated the impact of incorporating spices on the consumer acceptability and antioxidant activity of probiotic yogurt. Their findings indicated that probiotic yogurts containing *Lactobacillus acidophilus* (strain 5) and *Bifidobacterium lactis* (strain 12), supplemented with cinnamon, cardamom, and nutmeg, exhibited favorable sensory properties. Additionally, the spices were found to enhance the survival and viability of probiotic microorganisms (Illupapalayam et al. 2014).

Building on these insights, the present study aims to evaluate the effects of incorporating an aqueous extract of *R. canina* on the antioxidant activity, sensory evaluations, and physicochemical properties of yogurt. By exploring the potential of *R. canina* as a functional ingredient, this research seeks to contribute to the development of yogurt with enhanced health benefits and improved sensory appeal.

## 2 Materials and Methods

In this study, the impact of *R. canina* fruit extract on the antioxidant, physicochemical, and sensory properties of yogurt was investigated over a 3‐week refrigerated storage period, with analyses conducted on Days 1, 7, 14, and 21. All experiments were performed in triplicate to ensure statistical reliability. The study compared yogurt formulations without the extract (serving as the control group) against yogurts supplemented with *R. canina* extract at concentrations of 0.5%, 1%, and 1.5% (w/v) to systematically evaluate the influence of varying extract concentrations on the functional and sensory characteristics of yogurt during storage.

### 2.1 Extraction of *R. canina*

A total of 250 g of powdered samples was mixed with 1250 mL of distilled water as the solvent and agitated in a shaker for 2 h. The mixture was then centrifuged at 3000 rpm for 10 min to separate the solid residues. The resulting supernatant was freeze‐dried to obtain the extract. The final product was stored in amber‐colored jars and refrigerated until further use.

### 2.2 Preparation of Yogurt

To prepare the yogurt, 60 g of powdered milk were added to four liters of pasteurized and homogenized preheated milk to achieve a dry matter content of 12%. The milk was heated until boiling, maintained for 10 min, and then cooled to 42°C. At this temperature, yogurt starter cultures (obtained from Dalton Biotechnology, Italy) were introduced. *R. canina* extract was incorporated at three concentrations: 0.5%, 1%, and 1.5%, while the control sample was prepared without the extract (Table 1). The mixture was thoroughly homogenized and distributed into 100 mL sterile jars. The samples were then incubated at 42°C for 42 h until a pH of 4.6 was achieved. Following incubation, the yogurt samples were stored at 4°C until further analysis (Hasan‐Nejad et al. 2005; Ramchandran and Shah 2010).

**TABLE 1:** Different treatments in this study.

Row	Treatments	Description
1	Control	Yogurt
2	0.5%	Yogurt + 0.5% extract of *Rosa canina*
3	1.0%	Yogurt + 1.0% extract of *Rosa canina*
4	1.5%	Yogurt + 1.5% extract of *Rosa canina*

### 2.3 Phenolic Compounds

The quantification of phenolic compounds was performed using the Folin–Ciocalteu colorimetric method. 10 g of yogurt was centrifuged at 5000 rpm for 15 min, and 0.1 mL of the supernatant was transferred into separate test tubes. To each tube, 2 mL of sodium carbonate solution and 0.1 mL of Folin–Ciocalteu reagent were added. After a 2 h incubation period, the absorbance of the samples was measured at 750 nm using a spectrophotometer, with a blank consisting of 2 mL of sodium carbonate and 0.1 mL of Folin–Ciocalteu reagent as the reference (Abolfazl et al. 2020; Kamkar et al. 2021). The total phenolic content, expressed as milligrams of Gallic acid equivalents per kilogram of yogurt (mg GAE/kg), was determined using a calibration curve (Vasco et al. 2008).

### 2.4 DPPH

To assess antioxidant activity, 0.1 mL of each sample was mixed with 3 mL of 0.004% DPPH (2,2‐diphenyl‐1‐picrylhydrazyl) solution and thoroughly homogenized. After incubating the mixture for 30 min in the dark, the absorbance was measured at 517 nm using a spectrophotometer. The percentage of free radical scavenging activity was calculated using the following formula (Gheitanchi et al. 2021; Kamkar et al. 2021):

$$\begin{array}{c} \text{DPPH Scavenging Activity}\;\left(\%\right)=\\ \left(\left(A_{\text{control}}–A_{\text{sample}}\right)/A_{\text{control}}\right)\times 100 \end{array}$$

where A_control_ represents the absorbance of the DPPH solution without the sample, and A_sample_ represents the absorbance of the DPPH solution with the sample.

By the calibration curve of BHT concentration and inhibition percentage, the amount of antioxidant activity of the samples was calculated in terms of mg equivalent of BHT per kg of the sample (Gad and El‐Salam 2010).

### 2.5 ABTS

To prepare ABTS, 7.4 mM ABTS powder was added to 2.6 mM Potassium persulfate in phosphate buffer. The mixture was placed in a dark place at room temperature for 12–16 h before consumption. 0.1 mL of centrifuged yogurt was added to 2 mL of the prepared ABTS solution. After 5 min, the absorbance was measured by a spectrophotometer at 734 nm. The percentage of free radical scavenging was calculated from the following formula:

$$\begin{array}{c} \text{ABTS Scavenging Activity}\;\left(\%\right)=\\ \left(\left(A_{\text{control}}–A_{\text{sample}}\right)/A_{\text{control}}\right)\times 100 \end{array}$$

where A_control_ represents the absorbance of the ABTS solution without the sample, and A_sample_ represents the absorbance of the ABTS solution with the sample.

By the calibration curve of BHT concentration and inhibition percentage, the amount of antioxidant activity of the samples was calculated in terms of mg equivalent of BHT per kg of the sample (Vasco et al. 2008).

### 2.6 Acidity and pH

The pH of the yogurt samples was measured using a calibrated pH meter. Prior to measurement, the electrode was rinsed with a neutral buffer solution and then immersed directly into the samples (Abolfazl et al. 2020; Kamkar et al. 2021). The pH value displayed on the device was recorded as the pH of the yogurt.

For acidity determination, the method outlined in the Iranian National Standard (ISIRI Number 2852) was followed. Ten grams of yogurt were mixed with 90 mL of cooled, boiled water. Subsequently, 0.5 mL of phenolphthalein indicator was added to the mixture. The solution was then titrated with 0.1 N sodium hydroxide (NaOH) until a pale pink endpoint was achieved, which persisted for at least 5 s. The acidity was calculated based on the volume of NaOH required to reach the endpoint.

$$\%\text{Acidity}=1000\times N\;\left(V-V^{\prime }\right)/W$$

where: *N*, normality of the titrant; V, volume of titrant used for the sample (mL); V′, volume of titrant used for the blank (mL); W, weight of the sample (g).

### 2.7 Syneresis

Twenty grams of yogurt samples were put in a refrigerated centrifuge at 4000 rpm for 15 min. The supernatant was separated and weighed. By the ratio of its weight to the initial weight of yogurt, the percentage of dehydration was reported (Ramchandran and Shah 2010).

$$\text{Syneresis rate}=\left(\left(\mathrm{mL}\right)\;\text{supernatant}\right)/\left(\left(\mathrm{mL}\right)\;\text{initial sample}\right)$$

### 2.8 Sensory Evaluation

Sensory evaluation of yogurt samples was performed by five trained volunteers (females, aged 22–30 years old). Panelists had previous experience with dairy sensory analysis and underwent a short orientation session on using the 5‐point hedonic scale on the 21st day of the study according to standards 695 and 4940 (ISIRI Number 695, 4990). The samples were evaluated in terms of appearance, taste, texture, and general acceptance. Finally, the overall acceptance of the yogurt samples was evaluated using a 5‐point hedonic scale. The sensory characteristics were graded as follows:

- Score 5: Complete conformity with the sensory characteristics specified in the relevant standard.
- Score 4: Minimal deviation from the sensory characteristics outlined in the relevant standard.
- Score 3: Significant deviation from the sensory characteristics defined in the relevant standard.
- Score 2: High deviation from the sensory characteristics specified in the relevant standard.
- Score 1: Very high deviation from the sensory characteristics determined in the relevant standard.

This scoring system was used to assess the degree of alignment between the sensory properties of the yogurt samples and the established standards (Siró et al. 2008).

### 2.9 Statistical Analysis

The collected data were analyzed using R software (4.3.2). The one‐way analysis of variance (ANOVA) was used to determine the statistically significant difference among treatments for chemical analysis (followed by Tukey's post hoc test). The significance level was considered 0.05. The sensory evaluation of samples was analyzed using the Kruskal–Wallis nonparametric statistical test, and the Mann–Whitney test was used for comparison between different groups.

## 3 Results

Table 2 presents the descriptive statistical analysis of the physicochemical characteristics of all samples (including all treatments) during 3 weeks of refrigerated storage.

**TABLE 2:** Descriptive statistics of physicochemical characteristics of all yogurt samples.

	Mean	SD	Min	Max
Phenolic content (mg GAE/kg)	525.74	276.74	142.22	828.53
DPPH (mg BHA eq/kg)	1010.43	680.24	43.87	2207.161
ABTS (mg BHA eq/kg)	774.84	588.10	46.35	1788.7485
Acidity (g/L)	6.54	1.08	5.05	9.24
pH	4.22	0.08	4.04	4.44
Syneresis	47.30	4.29	39.80	59.10
Sensory evaluations	—	—	1	5

### 3.1 Total Phenolic Compounds

**FIGURE 1:**
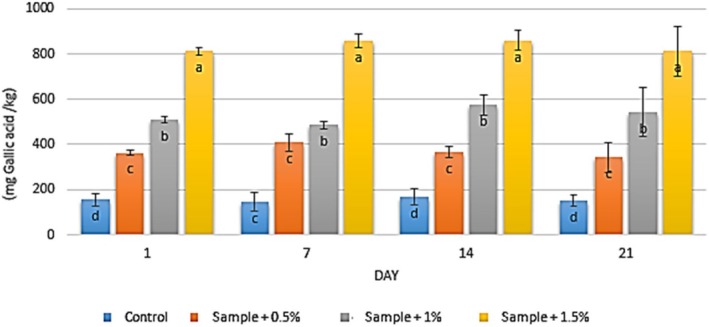
The effect of aqueous extract of *Rosa canina* on the total phenolic content in the studied groups during 3 weeks of refrigerated storage (Days 1, 7, 14, and 21). Values represent mean ± SD and the ones followed by different lowercase letters on the same days are significantly different. Statistical significance was determined using one‐way ANOVA followed by Tukey's post hoc test. Different letters indicate significant differences at *α* = 0.05.

The total phenolic content in the yogurt samples was initially measured at 154.30 mg Gallic acid equivalents per kilogram (mg GAE/kg), which decreased to 150.67 mg GAE/kg in the control group after 21 days of refrigerated storage (Figure 1). The results demonstrated that the incorporation of *R. canina* extract at varying concentrations significantly influenced the total phenolic content of the yogurt samples compared to the control group (*p* < 0.05). A linear and direct correlation was observed between the concentration of the extract and the total phenolic content across all storage intervals. The addition of 0.5% aqueous *R. canina* extract elevated the total phenolic content, with values ranging from 341.67 to 408 mg GAE/kg over the storage period. A dose‐dependent increase in total phenolic content was evident, with higher extract concentrations resulting in significantly greater phenolic levels (*p* < 0.05). Specifically, increasing the extract concentration to 1.5% raised the total phenolic content from 154.3 mg GAE/kg (control) to 810.67 mg GAE/kg. However, storage time did not exhibit a significant effect on the total phenolic content of the samples (*p* > 0.05).

### 3.2 DPPH

**FIGURE 2:**
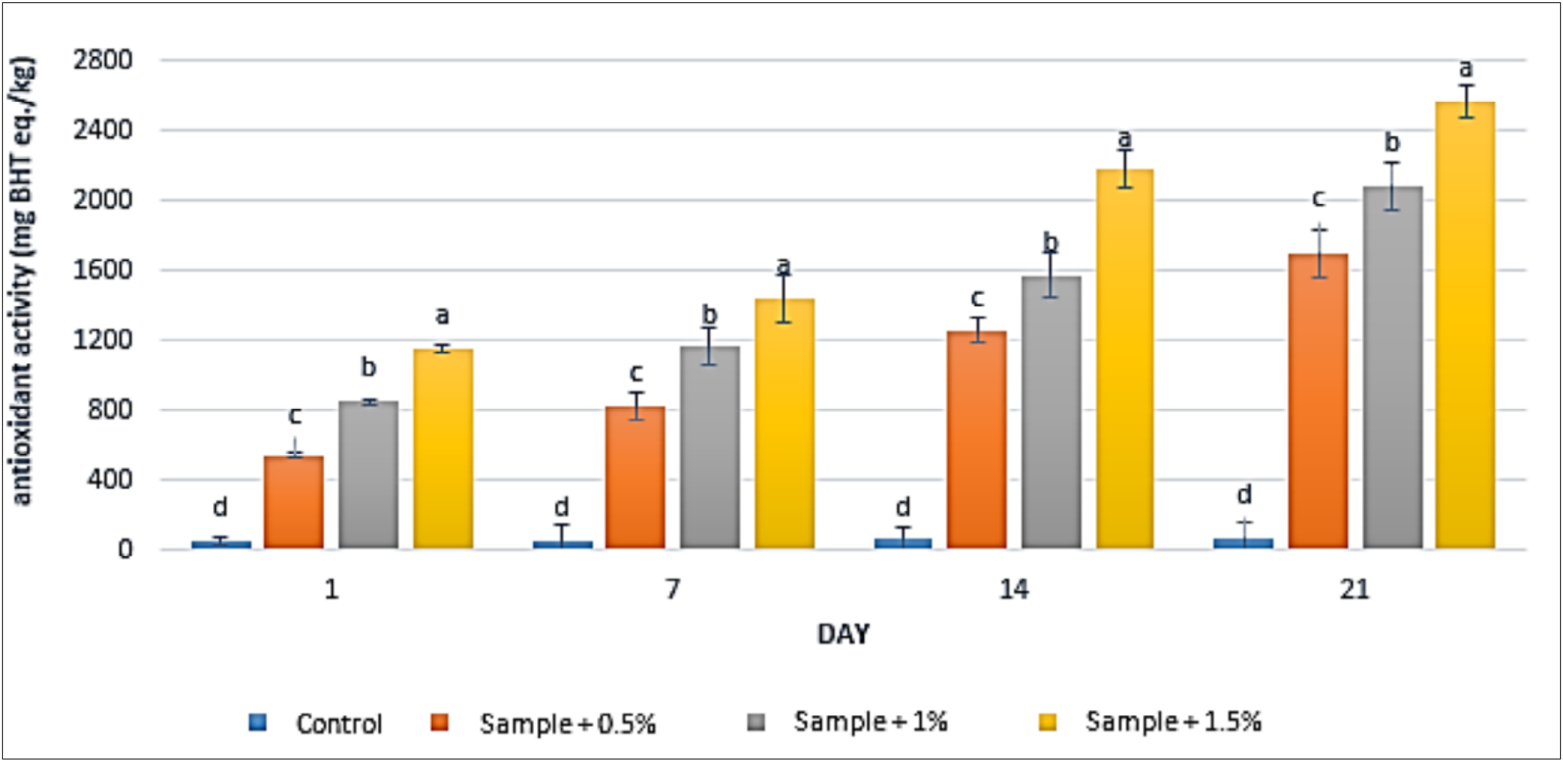
The effect of aqueous extract of *Rosa canina* on DPPH in the studied groups during 3 weeks of refrigerated storage (Days 1, 7, 14, and 21). Values represent mean ± SD and the ones followed by different lowercase letters on the same days are significantly different. Statistical significance was determined using one‐way ANOVA followed by Tukey's post hoc test. Different letters indicate significant differences at *α* = 0.05.

The results of antioxidant activity measurements in yogurt samples containing varying concentrations of *R. canina* extract (0%, 0.5%, 1%, and 1.5%) during 21 days of refrigerated storage are presented in Figure 2. The DPPH assay revealed a strong positive correlation between the antioxidant activity and the concentration of *R. canina* extract in the yogurt samples. The inclusion of *R. canina* extract at all tested concentrations significantly enhanced the antioxidant activity of the yogurt samples compared to the control group (*p* < 0.05). In the control group, antioxidant activity increased from 54.67 to 76 mg butylated hydroxytoluene equivalents per kilogram (mg BHT eq./kg) between the first and 21st day of storage. For the sample containing 0.5% extract, antioxidant activity rose from 545 to 1699.67 mg BHT eq./kg, while in the sample with 1% extract, it increased from 853 to 2082 mg BHT eq./kg. The highest antioxidant activity was observed in the sample containing 1.5% extract, which increased from 1157 to 2570.67 mg BHT eq./kg over the same period. Additionally, prolonged storage time significantly augmented the antioxidant activity of the yogurt samples (*p* < 0.05).

### 3.3 ABTS

**FIGURE 3:**
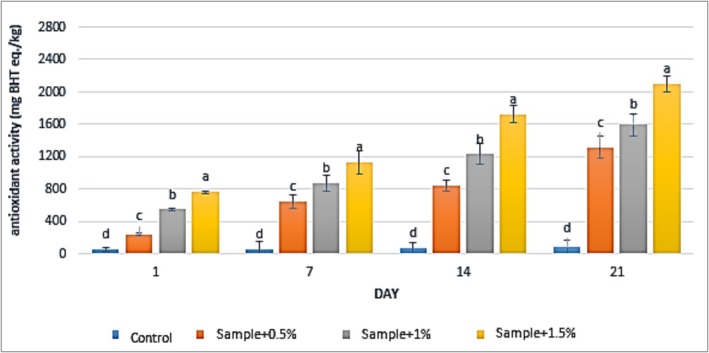
The effect of aqueous extract of *Rosa canina* on ABTS in the studied groups during 3 weeks of refrigerated storage (Days 1, 7, 14, and 21). Values represent mean ± SD and the ones followed by different lowercase letters on the same days are significantly different. Statistical significance was determined using one‐way ANOVA followed by Tukey's post hoc test. Different letters indicate significant differences at *α* = 0.05.

The results of antioxidant activity measurements in yogurt samples containing varying concentrations of *R. canina* extract (0%, 0.5%, 1%, and 1.5%) during 21 days of refrigerated storage are presented in Figure 3. Consistent with the findings from the DPPH assay, a strong positive correlation was observed between the antioxidant activity and the concentration of *R. canina* extract in the yogurt samples. The addition of *R. canina* extract at all tested levels significantly increased the antioxidant activity of the yogurt samples compared to the control group (*p* < 0.05). In the control group, antioxidant activity increased from 54 to 76.33 mg butylated hydroxytoluene equivalents per kilogram (mg BHT eq./kg) over the storage period. For the yogurt containing 0.5% extract, antioxidant activity rose from 239 to 1312.33 mg BHT eq./kg, while in the sample with 1% extract, it increased from 547 to 1592 mg BHT eq./kg. The highest antioxidant activity was observed in the yogurt containing 1.5% extract, which increased from 760.33 to 2091.33 mg BHT eq./kg during the same period. Furthermore, extended storage time significantly enhanced the antioxidant activity of the yogurt samples (*p* < 0.05).

### 3.4 Acidity

**FIGURE 4:**
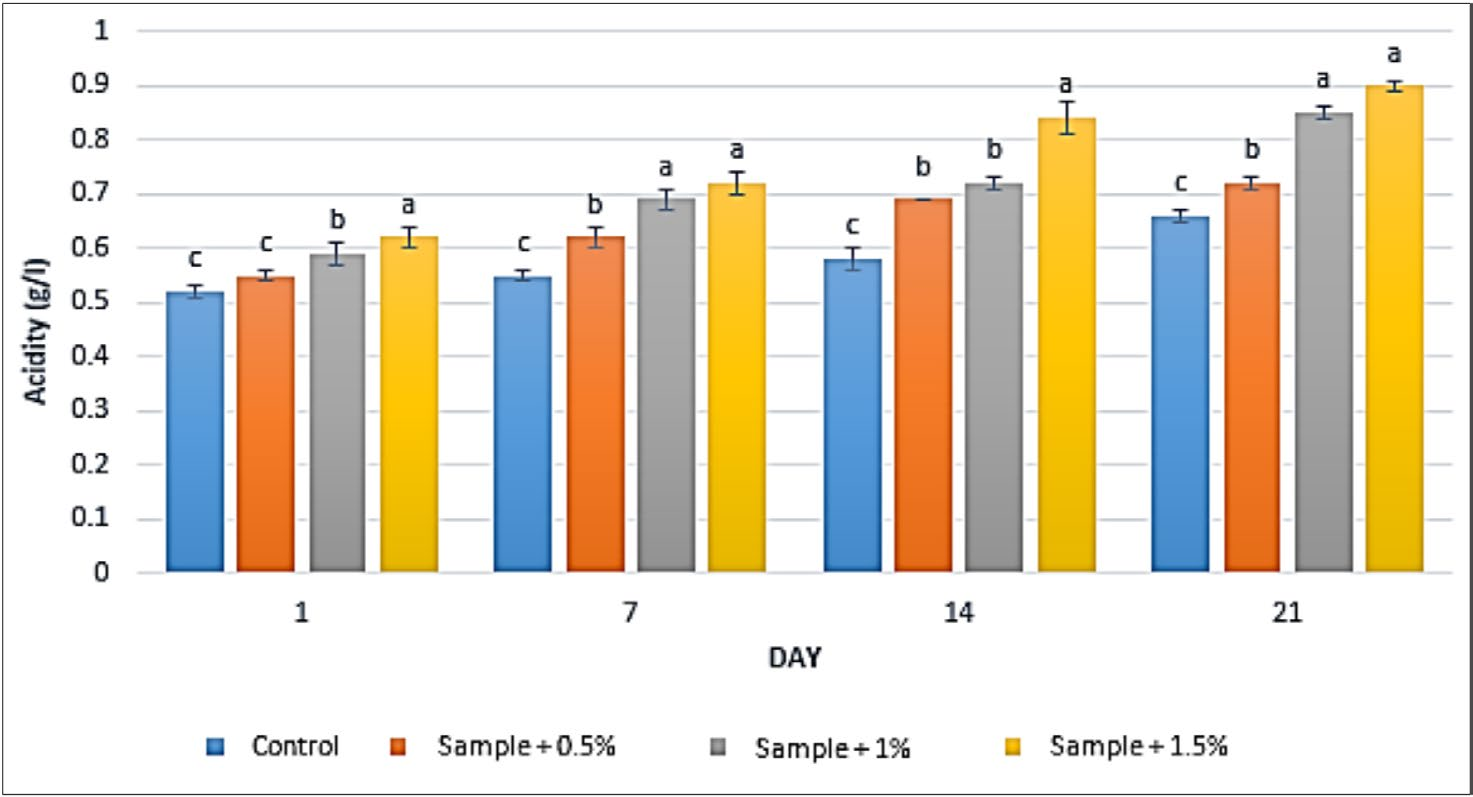
The effect of aqueous extract of *Rosa canina* on acidity in the studied groups during 3 weeks of refrigerated storage (Days 1, 7, 14, and 21). Values represent mean ± SD, and the ones followed by different lowercase letters on the same days are significantly different. Statistical significance was determined using one‐way ANOVA followed by Tukey's post hoc test. Different letters indicate significant differences at *α* = 0.05.

The results of acidity measurements in yogurt samples containing varying concentrations of *R. canina* extract (0%, 0.5%, 1%, and 1.5%) during 21 days of refrigerated storage are presented in Figure 4. At the beginning of the study (Day 1), the acidity of the control group was 0.52 g/L, which increased to 0.66 g/L by the end of the storage period. The addition of *R. canina* extract at different concentrations significantly influenced the acidity of the yogurt samples compared to the control group (*p* < 0.05). Incorporating 1.5% *R. canina* extract into yogurt increased the initial acidity from 0.52 g/L (control) to 0.62 g/L. Furthermore, the results demonstrated that prolonged storage time led to a significant increase in acidity across all yogurt samples. Specifically, the acidity levels (between Days 1 and 21) increased in the yogurt containing 0.5% extract from 0.55 to 0.72 g/L, in the yogurt with 1% extract from 0.59 to 0.85 g/L, and in the yogurt containing 1.5% extract from 0.62 to 0.90 g/L.

### 3.5 pH

**FIGURE 5:**
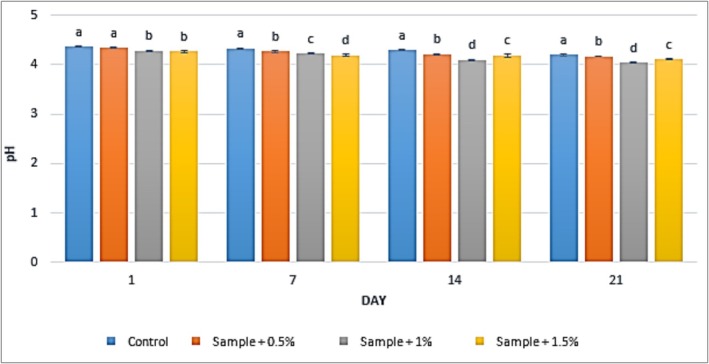
The effect of aqueous extract of *Rosa canina* on pH in the studied groups during 3 weeks of refrigerated storage (Days 1, 7, 14, and 21). Values represent mean ± SD and the ones followed by different lowercase letters on the same days are significantly different. Statistical significance was determined using one‐way ANOVA followed by Tukey's post hoc test. Different letters indicate significant differences at *α* = 0.05.

The results of pH measurements in yogurt samples containing varying concentrations of *R. canina* extract (0%, 0.5%, 1%, and 1.5%) during 21 days of refrigerated storage, including the control treatment, are presented in Figure 5. The findings indicate that the addition of *R. canina* extract led to a reduction in the pH of the yogurt samples. In the control group, the pH decreased from 4.36 to 4.2 over the storage period (Days 1–21). For the yogurt containing 0.5% extract, the pH ranged from 4.34 to 4.16, while in the 1% extract group, it decreased from 4.28 to 4.04. In the yogurt samples containing 1.5% *R. canina* extract, the pH varied from 4.27 to 4.11. The changes in pH across different storage intervals demonstrated significant variations with increasing concentrations of *R. canina* extract (*p* < 0.05).

### 3.6 Syneresis

**FIGURE 6:**
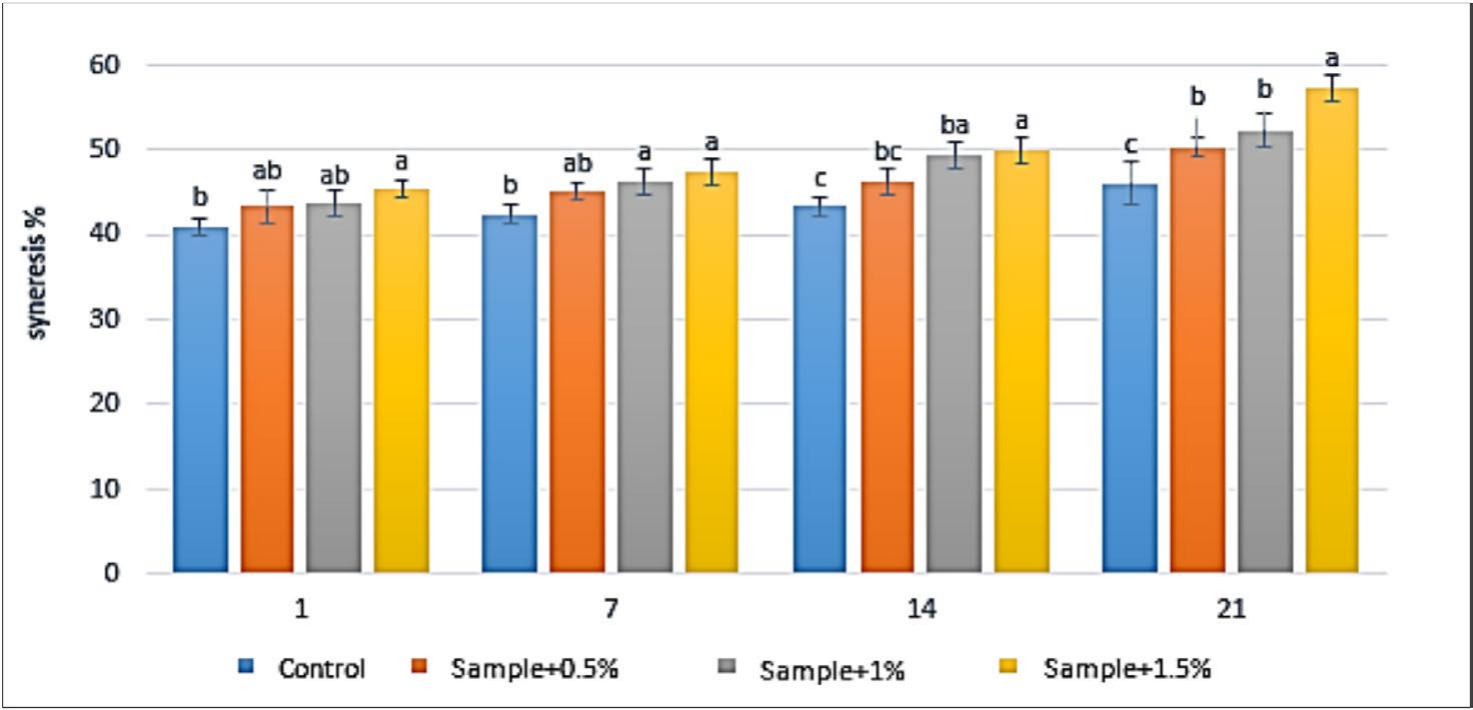
The effect of aqueous extract of *Rosa canina* on the syneresis in the studied groups during 3 weeks of refrigerated storage (Days 1, 7, 14, and 21). Values represent mean ± SD, and the ones followed by different lowercase letters on the same days are significantly different. Statistical significance was determined using one‐way ANOVA followed by Tukey's post hoc test. Different letters indicate significant differences at *α* = 0.05.

The results of syneresis measurements in yogurt samples containing varying concentrations of *R. canina* extract (0%, 0.5%, 1%, and 1.5%) during 21 days of refrigerated storage, including the control treatment, are presented in Figure 6. The findings indicate that increasing the concentration of *R. canina* extract led to a rise in syneresis levels in the yogurt samples across all storage periods. On Day 1, a significant difference (*p* < 0.05) was observed between the syneresis rate of the control yogurt sample and the sample containing 1.5% extract. On Days 7, 14, and 21, statistically significant differences (*p* < 0.05) were observed between all extract‐containing samples and the control group. In the control group, the syneresis rate increased from 41% to 46% between Days 1 and 21. For the yogurt containing 0.5% extract, syneresis rose from 43.3% to 50.3%, while in the 1% extract group, it increased from 43.3% to 52.3%. In the yogurt samples containing 1.5% *R. canina* extract, syneresis increased from 45.3% to 57.3% over the same period.

### 3.7 Sensory Evaluation

**FIGURE 7:**
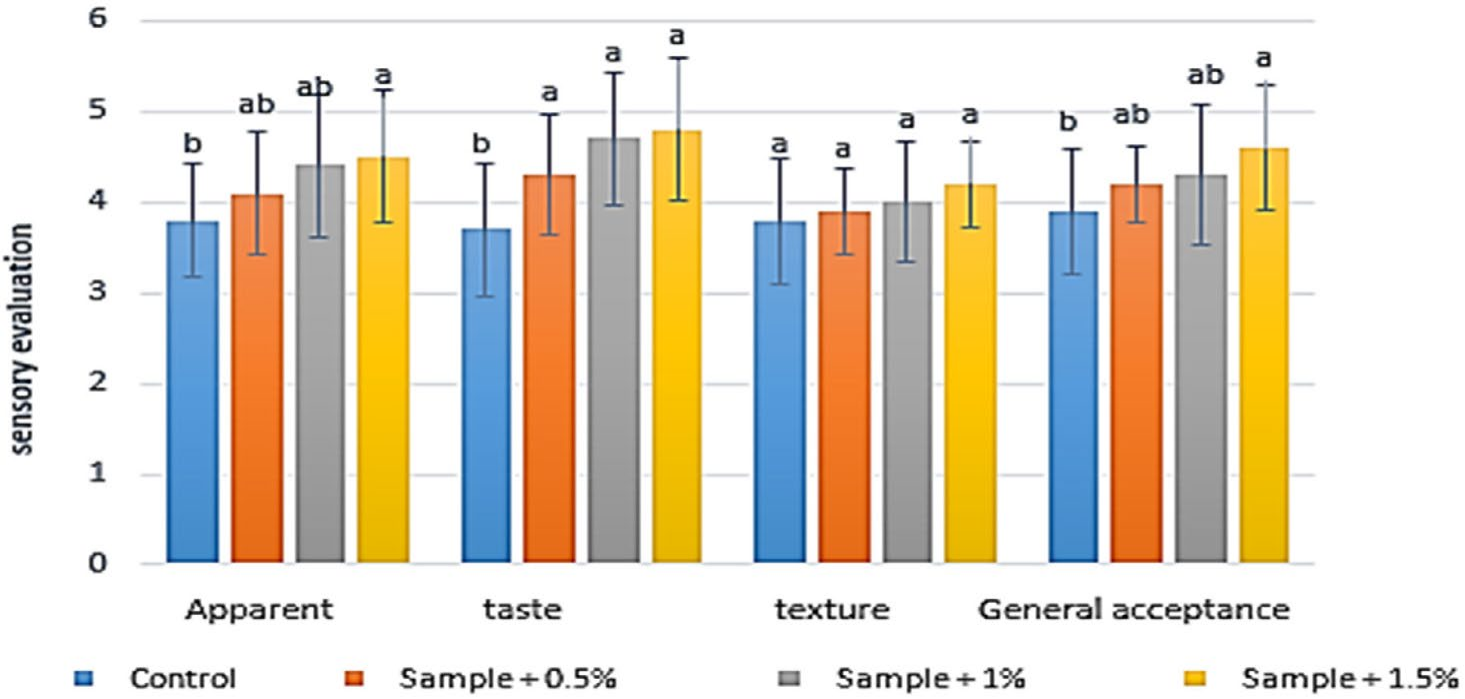
The effect of aqueous extract of *Rosa canina* on the sensory evaluation in the studied groups after 3 weeks of refrigerated storage. Values represent mean ± SD and the ones followed by different lowercase letters on the same days are significantly different. Statistical significance was determined using the Kruskal–Wallis test. Different letters indicate significant differences at *α* = 0.05.

The results of comparing the average effect of different concentrations of *R. canina* extract on the sensory and physical characteristics of yogurt are presented in Figure 7. The findings indicate that the addition of varying percentages of *R. canina* extract enhanced the quality attributes of the yogurt. Regarding appearance, increasing the extract concentration from 0.5% to 1.5% improved the score from 3.8 (control) to 4.5 (1.5% extract). Similarly, the taste of the yogurt improved with higher extract concentrations, with the taste score increasing from 3.7 (control) to 4.6 (1.5% extract). However, no significant differences were observed in taste scores among samples containing 0.5%, 1%, and 1.5% extract. In terms of texture, the addition of *R. canina* extract did not significantly influence the texture scores of the yogurt samples. Furthermore, the overall sensory score of the yogurt increased with higher extract concentrations, rising from 3.9 (control) to 4.6 (1.5% extract).

### 3.8 Principal Component Analysis

**FIGURE 8:**
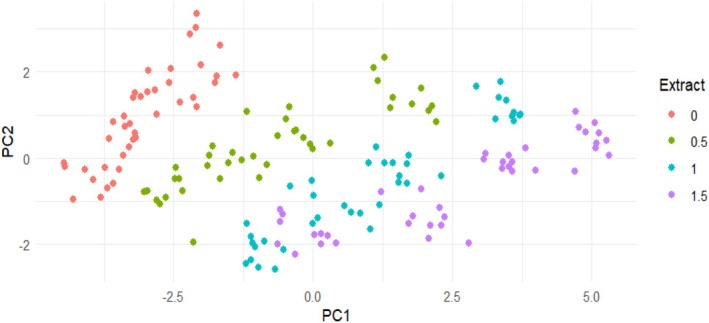
Principal component analysis (PCA) plot illustrating the effect of different *Rosa canina* extract concentrations (0%, 0.5%, 1%, and 1.5%) on yogurt characteristics. The first two principal components (PC1 and PC2) explain the variation in the datasets, grouping samples based on extract levels.

The PCA plot (Figure 8) shows the distribution of yogurt samples based on their physicochemical properties, influenced by different *R. canina* extract concentrations. The first principal component (PC1) separates samples primarily along the extract concentration gradient, suggesting that extract levels significantly contribute to variability in the dataset.

Samples without extract (0%) cluster on the left, while those with higher extract levels (1% and 1.5%) shift toward the right. This separation indicates that increasing extract concentrations induce notable changes in yogurt traits, likely due to variations in phenolic content, antioxidant capacity (DPPH, ABTS), acidity, pH, and syneresis. The clustering pattern implies a progressive shift in the yogurt's characteristics with increasing extract concentration.

### 3.9 Correlation Between Physicochemical Properties and Sensory Attributes

**FIGURE 9:**
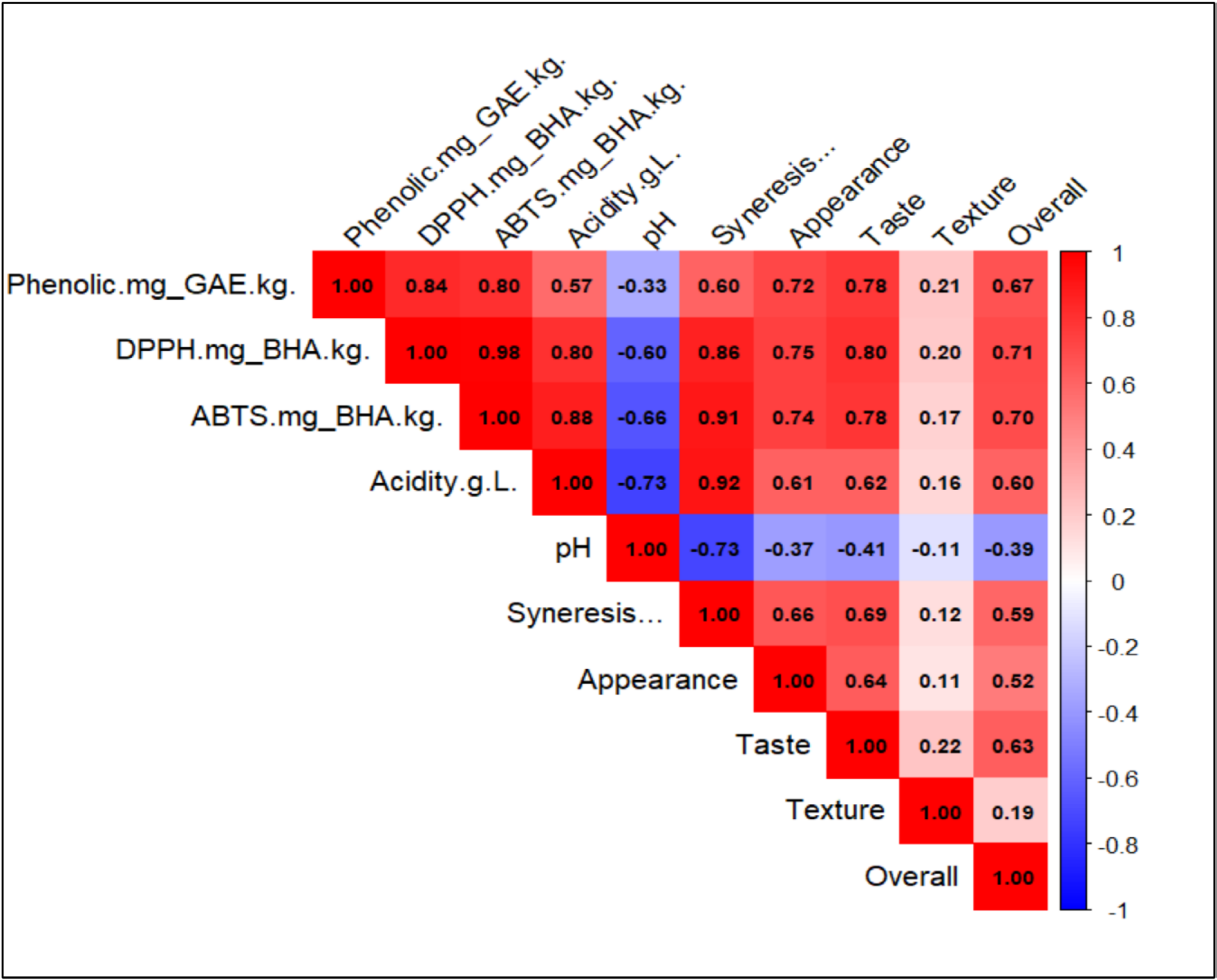
Correlation matrix illustrating the relationships between physicochemical and sensory traits of yogurt samples with different *Rosa canina* extract concentrations. Positive correlations are shown in red, while negative correlations are in blue.

The correlation matrix (Figure 9) reveals significant relationships between the yogurt's physicochemical properties and sensory attributes. Notably, phenolic content, DPPH, and ABTS exhibit strong positive correlations (*r* > 0.80), indicating that antioxidant properties are interrelated. These antioxidant traits also show moderate to strong positive correlations with syneresis (*r* = 0.60 to 0.92), suggesting that increased extract levels may influence whey separation. Acidity and pH are inversely correlated (*r* = −0.73), as expected, confirming that higher acidity leads to a lower pH. Interestingly, pH is negatively correlated with syneresis (*r* = −0.73) and overall acceptability (*r* = −0.39), implying that lower pH (higher acidity) affects both physical stability and consumer preference. Among sensory traits, appearance, taste, and texture positively correlate with overall acceptability (*r* = 0.52 to 0.63), indicating that visual and textural properties significantly influence consumer perception. However, phenolic content and antioxidant capacity (DPPH, ABTS) show weaker correlations with taste (*r* ≈0.20), suggesting that while *R. canina* extract enhances antioxidant properties, it may not strongly impact taste perception. These findings highlight that increasing *R. canina* extract affects both chemical composition and sensory quality, emphasizing a trade‐off between antioxidant benefits and product stability.

## 4 Discussion

A recent study has reported the total phenolic compound content in *R. canina* fruit to be approximately 96 mg GA/kg (Ercisli 2007). Demir et al. (2014) further analyzed the bioactive composition of *R. canina* fruit, highlighting its richness in phenolic and flavonoid compounds, with their concentrations varying depending on species and cultivation conditions. Similarly, Zhang et al. (2019) investigated the impact of *Moringa* plant extract on the quality and bioactive properties of yogurt, reporting a significant increase in phenolic compound content following the addition of *Moringa* leaf extract. This increase was attributed to the inherently high phenolic content of *Moringa* extract. Our findings align with these previous observations.

Antioxidant compounds are characterized by their ability to donate hydrogen atoms or electrons, leading to a color change in the DPPH assay from purple to yellow (Sanchez‐Segarra et al. 2000; Mosiyani et al. 2017). Fattahi et al. (2012) examined the phenolic content and antioxidant capacity of *R. canina* fruit and identified a significant positive correlation between phenolic content and antioxidant activity, indicating that higher phenolic concentrations corresponded to greater antioxidant potential. Consistently, our study demonstrated a significant increase in antioxidant capacity with increasing concentrations of *R. canina* extract.

Although the DPPH assay is widely used to assess the free radical scavenging activity of natural products and food matrices, it has limitations, particularly its inability to measure hydrophilic antioxidants. This is due to its solubility in organic solvents, such as alcohols, rather than aqueous solutions. In contrast, the ABTS assay is applicable to both hydrophilic and lipophilic antioxidants, making it a complementary method for assessing antioxidant potential. The ABTS assay is based on the reduction of a stable green‐blue radical to a colorless product (O'Sullivan et al. 2016). In agreement with our DPPH assay results, the increased bioactive compound content in our study was accompanied by enhanced antioxidant capacity as determined by the ABTS assay. Zhang et al. (2019) similarly observed an increase in antioxidant capacity (ABTS assay) in yogurt samples enriched with *Moringa* extract.

The denaturation of whey proteins plays a critical role in improving water‐holding capacity and reducing syneresis. However, as the whey protein‐to‐casein micelle ratio increases, syneresis may also increase due to the reduced availability of casein micelles, which are essential for gel formation. In our study, the addition of *R. canina* extract significantly increased syneresis in yogurt samples during storage. However, conflicting reports exist in the literature regarding the effect of plant extracts on syneresis. Lotfizade Dehkordi et al. (2013) found that *Sheng* plant extract, due to its inulin content, reduced syneresis relative to control samples. Similarly, Alirezalu et al. (2019) reported decreased syneresis in yogurt supplemented with sugar beet, spinach, and tomato extracts. Conversely, Amirdivani and Baba (2013) demonstrated that the incorporation of 2% Malaysian and Japanese green tea extracts led to increased syneresis over time. In our study, the incorporation of *R. canina* extract significantly increased syneresis in yogurt samples, particularly at higher concentrations. This phenomenon can be explained by the complex interactions between phenolic compounds and milk proteins. Phenolic molecules are known to bind to casein micelles through hydrogen bonding and hydrophobic interactions. While such interactions can sometimes improve gel strength, at higher levels they may disrupt the protein–protein associations that are essential for a stable yogurt gel. The binding of phenolic to caseins may interfere with the formation of a dense casein network, leading to reduced water‐holding capacity and consequently higher whey separation. Furthermore, the elevated acidity observed in extract‐containing yogurts likely contributed to this effect. Increased acidity accelerates casein micelle dissociation and promotes electrostatic repulsion between proteins, weakening the gel structure. When combined with the structural disruption caused by phenolic–protein interactions, this dual effect can markedly increase syneresis. Similar mechanisms have been reported in other plant‐enriched yogurts where high levels of polyphenols compromised gel integrity (Amirdivani and Baba 2013). Another possible factor is the oxidative activity of certain phenolic compounds. Although primarily antioxidants, phenolics can act as pro‐oxidants in the presence of proteins and transition metals, leading to protein modification and further destabilization of the gel matrix (Jakobek 2015). This may explain why yogurt samples with higher extract concentrations (1.0% and 1.5%) exhibited more pronounced whey separation compared to the control. From a practical perspective, increased syneresis negatively affects product appearance and consumer acceptance, as whey separation is often perceived as a defect in yogurt. Therefore, while *R. canina* extract improves antioxidant properties and sensory scores for flavor and appearance, its destabilizing impact on gel structure at higher concentrations highlights the importance of optimizing extract levels to balance health benefits with desirable textural quality (Amirdivani and Baba 2013).

In the present study, *R. canina* fruit extract resulted in increased acidity and decreased pH in yogurt samples. Over the storage period, acidity continued to increase, consistent with the findings of Mosiyani et al. (2017), who reported that the inclusion of plant extracts in yogurt significantly enhanced acidity and lowered pH compared to control samples. The increased acidity observed in these studies is likely due to the enhanced metabolic activity of lactic acid bacteria, which produce organic acids during milk fermentation.

The overall quality of fermented dairy products is largely determined by sensory characteristics such as taste, texture, and appearance (Smith 2003; Lotfizade Dehkordi et al. 2013). In our study, the addition of *R. canina* extract improved various sensory attributes. The sweet–sour flavor and the desirable light red color of the yogurt contributed to enhanced quality perception. A comparison of qualitative traits revealed that yogurt containing *R. canina* extract received the highest taste and acceptance scores, with increasing extract concentration correlating with higher acceptance ratings. The improvement in aroma was likely due to the presence of aromatic compounds in *R. canina* extract. However, no significant differences in texture were observed between the control and extract‐containing samples, which may be attributed to the syneresis effect. Zhang et al. (2019) reported a decline in overall acceptability with increasing *Moringa* extract concentration in yogurt, highlighting the importance of optimizing extract levels to maintain desirable sensory attributes.

## 5 Conclusion

In this study, the effects of incorporating an aqueous extract of *R. canina* on the antioxidant activity, sensory attributes, and physicochemical properties of yogurt were systematically evaluated. The results demonstrated that the addition of *R. canina* extract significantly enhanced the antioxidant properties and phenolic content of yogurt. However, syneresis levels increased with higher concentrations of the extract. Additionally, the study revealed that increasing the extract concentration led to a rise in acidity and a corresponding decrease in pH over the storage period. Given the positive impact of *R. canina* fruit extract on the sensory properties of yogurt, its incorporation into dairy products, such as yogurt, is recommended as a practical and effective strategy to enhance both the functional and sensory qualities of these products. This approach not only improves the nutritional profile of yogurt but also contributes to promoting consumer health. Future research should explore optimization of extract concentration to minimize syneresis, as well as investigating microencapsulation or stabilizers to improve texture and consumer acceptance.

## Author Contributions

**Shokoofeh Hosseini:** investigation (equal). **Fatemeh Gheitanchi:** software, writing – original draft (equal). **Abolfazl Kamkar:** project administration (equal). **Sina Sakhaeifar:** software, writing – review, and editing (equal).

## Ethics Statement

This study does not involve any human or animal testing. The sensory evaluation protocol was approved by the Committee of the Faculty of Veterinary Medicine, University of Tehran.

## Conflicts of Interest

The authors declare no conflicts of interest.

## Data Availability

The data that support the findings of this study are available from the corresponding author upon reasonable request.
